# Congenital ulcerated nodule: self-healing Langerhans cell histiocytosis^⋆^

**DOI:** 10.1016/j.abd.2021.12.012

**Published:** 2023-04-26

**Authors:** Tiago Fernandes Gomes, José Carlos Cardoso, Victoria Guiote, Felicidade Santiago

**Affiliations:** a Department of Dermatology, Centro Hospitalar de Leiria, Portugal; b Department of Dermatology, Centro Hospitalar e Universitário de Coimbra, Portugal

Dear Editor,

A full-term male newborn born by vaginal delivery, after an uncomplicated gestation, was observed at our department on the first day of life for a congenital cutaneous lesion. At physical examination, we observed on the left scapular region an 8 mm ulcerated nodule, with a central black crust, a bright elevated pink border, and a peripheral erythema (Fig. 1). Dermoscopy revealed a central reddish-black crust, with a rim of sparse red globules, and a pink border with fine white scale (Fig. 2). No other lesions were evident, and the remaining physical examination had no abnormalities. Eye red reflex and otoacoustic emissions screenings were normal. His family history was unremarkable. A punch biopsy was made.

**Figure 1:**
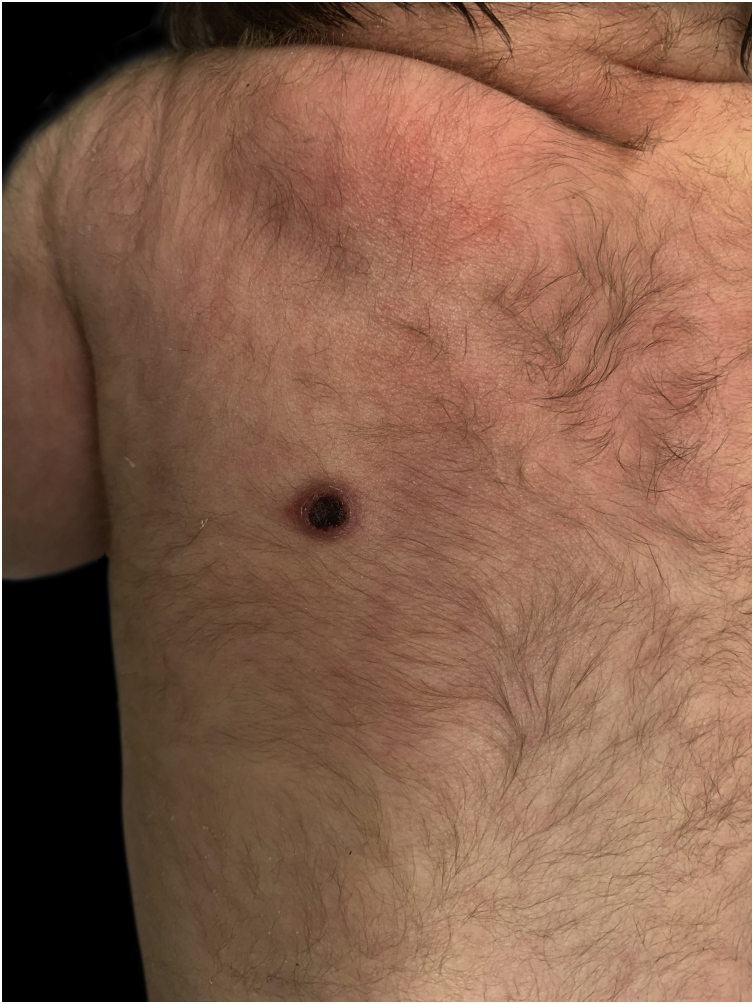
Ulcerated lesion with central crust and elevated pink border located on the left scapular region.

**Figure 2:**
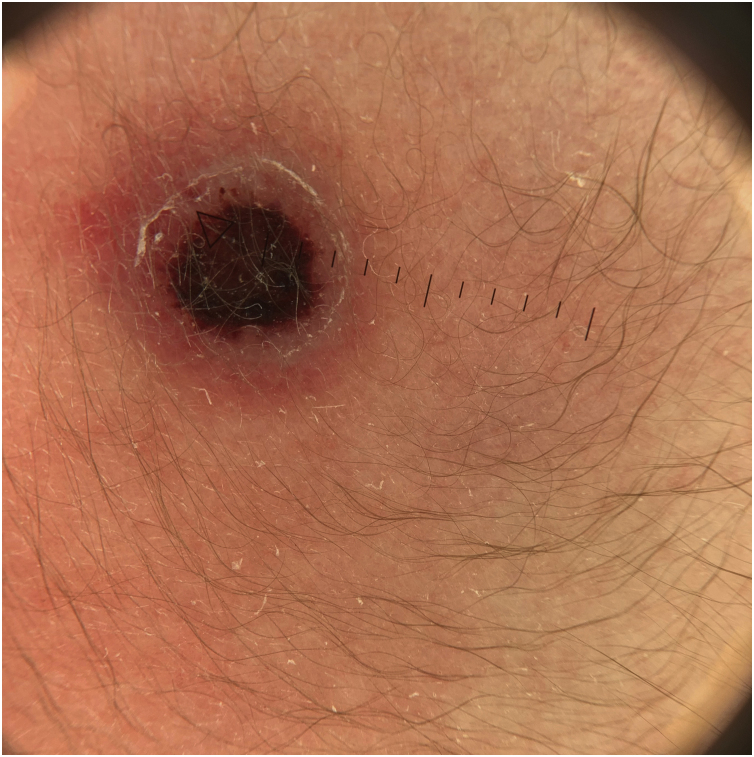
Dermoscopy with central reddish/black crust, a pink border with fine white scale and sparse red globules between the crust and the border.

Histopathology showed an ulcerated lesion with an infiltrate composed of large epithelioid cells with a kidney-shaped nucleus, with epidermotropism (Fig. 3). Mitotic figures were observed. The background infiltrate was composed of lymphocytes, plasma cells and a significant number of eosinophils (Fig. 4). Immunostaining was positive for CD1a (Fig. 5A), langerin (Fig. 5B), S100 protein and CD45, and negative for CD68 and CD34. A presumptive diagnosis of solitary congenital self-healing Langerhans cell histiocytoma was made. Complete Blood Count (CBC), Erythrocyte Sedimentation Rate (ESR), coagulation times, kidney and liver function tests and lactate dehydrogenase were within the normal range for the age. The newborn underwent chest radiography and abdominal ultrasound, which revealed no abnormalities.

**Figure 3:**
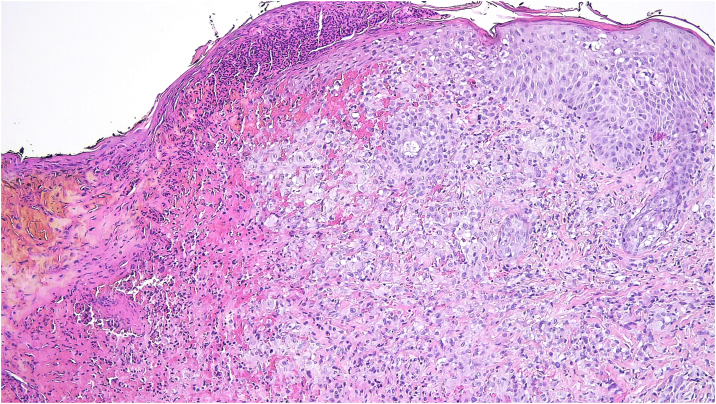
Ulcerated lesion with an infiltrate composed of epithelioid cells with kidney-shaped nucleus, with epidermotropism (Hematoxylin & eosin, ×100).

**Figure 4:**
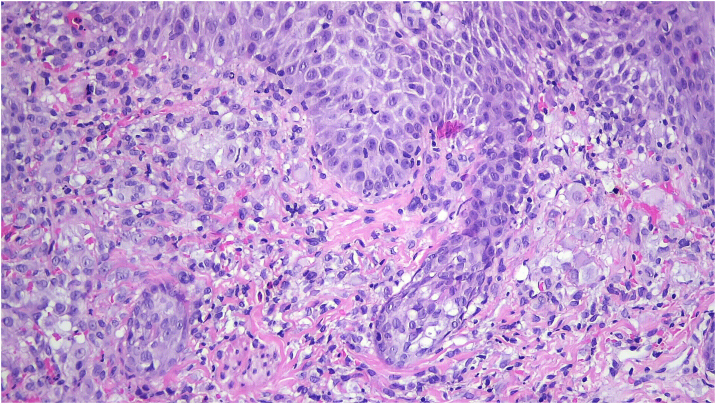
Infiltrate composed of epithelioid cells with kidney-shaped nucleus, accompanied by lymphocytes, plasma cells and a significant number of eosinophils (Hematoxylin & eosin, ×200).

**Figure 5:**
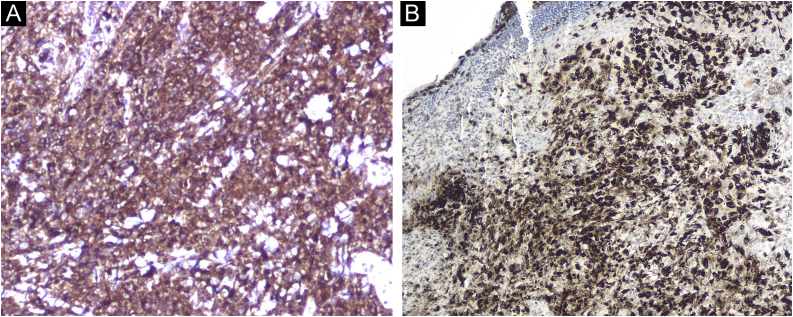
Positive immunostaining for CD1a (A, ×200) and langerin (B, ×100).

The infant maintains regular follow-ups. At 6 months appointment, no systemic involvement was noticed and there was a complete involution of the skin lesion. The overall well-appearing state of the neonate, lack of systemic signs, and spontaneous involution of the lesion, associated with the histopathology and immunohistochemistry findings, were compatible with congenital self-healing histiocytosis.

## Discussion

Langerhans cell histiocytosis (LCH) is a rare neoplasm, characterized by a pathological Langerhans cell proliferation.^1^ LCH can present as a single organ or multi-systemic involvement, with a wide spectrum of manifestations, ranging from self-resolving skin lesions to disseminated forms.^2^ The prognosis depends on the extent of systemic involvement, with the single-system disease having a good prognosis.^2^

Congenital self-healing Langerhans cell histiocytosis (CSHLCH) is a rare variant of LCH.^3^ It usually presents as multiple papules or nodules (from the “blueberry muffin baby” spectrum), but uncommonly a solitary lesion may occur.^3^, ^4^ LC histiocytoma (or solitary CSHLCH) is considered a unimodular or paucinodular CSHLCH variant. It presents as a single reddish nodule at birth or within the first weeks of life, that progresses to crusting and ulceration.^5^ Cutaneous lesions typically regress within a few months.^6^ Neonates are usually healthy, with no systemic involvement.^5^, ^6^ A recent retrospective review of 82 neonates showed complete involution of the lesion in all cases who have not been submitted to excision. Furthermore, the authors observed no progression, recurrence, or multisystemic involvement, with a median follow-up of 15 months.^6^

Histopathology is characterized by a dense dermal infiltrate composed of large cells with abundant eosinophilic cytoplasm and round-oval or kidney-shaped nucleus, accompanied by an infiltrate comprising lymphocytes and eosinophils.^4^, ^6^ Histopathological differences have been between solitary CSHLCH and multiple papular or nodular forms. In the former, there is a deeper infiltrate of the whole dermis and often extends to superficial subcutis, superficial ulceration or crusting, frequent parakeratosis and necrosis, and little or no epidermotropism. In solitary CSHLCH, there are large-sized cells, sometimes with “ground-glass” cytoplasm and numerous mitoses.^5^ Diagnosis is confirmed by positive immunostaining for CD1a, S100, and langerin, markers of Langerhans cells. Electron microscopy reveals the characteristic Birbeck granules,^6^ but usually is not needed.

There is some variability in dermoscopy reports of LCH, due to the clinical spectrum and different lesion stages. Reddish-lilac color of nodular lesions, with peripheral telangiectasias, violaceous lacunae and clods, and whitish areas may be present.^7^, ^8^ However, dermoscopy descriptions are sparse in the literature.

There are no specific guidelines for the management and follow-up of solitary CSHLCH. Initial work-up should include a complete physical examination (exclude other skin or mucosal lesions, lymphadenopathy, or hepatosplenomegaly), laboratory analysis (CBC, ESR, C-reactive protein, coagulation studies, hepatic and renal function tests, blood and urine osmolarities) and imaging exams (abdominal ultrasound, chest X-Ray, complete skeletal radiographs).^9^

In summary, solitary CSHLCH diagnosis should be raised in the presence of a single ulcerated congenital lesion and usually portends a good prognosis. CSHLCH diagnosis is made in retrospect after work-up and follow-up of these patients,^10^ showing a spontaneous resolution and absence of systemic involvement.

## Financial support

This research did not receive any specific grant from funding agencies in the public, commercial, or not-for-profit sectors.

## Authors' contributions

Tiago Fernandes Gomes: Data collection, analysis and interpretation; critical literature review; preparation and writing of the manuscript.

José Carlos Cardoso: Approval of the final version of the manuscript; data collection, analysis and interpretation; manuscript critical review.

Victoria Guiote: Approval of the final version of the manuscript; data collection, analysis, and interpretation; manuscript critical review.

Felicidade Santiago: Approval of the final version of the manuscript; data collection, analysis, and interpretation; intellectual participation in propaedeutic and/or therapeutic management of studied cases; manuscript critical review.

## Conflicts of interest

None declared.
